# Fabrication and compatibility evaluation of polycaprolactone/hydroxyapatite/collagen-based fiber scaffold for anterior cruciate ligament injury

**DOI:** 10.1039/d2ra07756f

**Published:** 2023-04-03

**Authors:** Aminatun, Faika Hanum S., Djoni Izak R., Sofijan Hadi, Tahta Amrillah, Che Azurahanim Che Abdullah

**Affiliations:** a Study Program of Physics, Department of Physics, Faculty of Science and Technology, Universitas Airlangga Surabaya Indonesia aminatun@fst.unair.ac.id; b Study Program of Biomedical Engineering, Department of Physics, Faculty of Science and Technology, Universitas Airlangga Surabaya Indonesia; c Study Program of Chemistry, Department of Physics, Faculty of Science and Technology, Universitas Airlangga Surabaya Indonesia; d Study Program of Nanotechnology Engineering, Faculty of Advanced Technology and Multidiscipline, Universitas Airlangga Surabaya Indonesia; e Institute of Nanoscience and Nanotechnology, Universiti Putra Malaysia 43400 UPM Serdang Selangor Malaysia

## Abstract

Knee injuries are musculoskeletal system injuries, including the Anterior Cruciate Ligament (ACL). ACL injuries are most common in athletes. This ACL injury necessitates biomaterial replacement. It is sometimes taken from the patient's tendon and a biomaterial scaffold is used. The use of biomaterial scaffolds as artificial ACLs remains to be investigated. The purpose of this study is to determine the properties of an ACL scaffold made of polycaprolactone (PCL)–hydroxyapatite (HA) and collagen with various composition variations of (50 : 45 : 5), (50 : 40 : 10), (50 : 35 : 15), (50 : 30 : 20), and (50 : 25 : 25) wt%. The scaffold was created using the electrospinning method with a voltage of 23 kV, a needle–collector distance of 15 cm, and a solution flow rate of 2 mL h^−1^. The average fiber diameter in all samples was less than 1000 nm. The model with the best characterization was PCL : HA : collagen with a weight-to-weight (wt%) ratio of 50 : 45 : 5 and an average fiber diameter of 488 ± 271 nm. The UTS and modulus of elasticity for braided samples were 2.796 MPa and 3.224 MPa, respectively, while the non-braided samples were 2.864 MPa and 12.942 MPa. The estimated time of degradation was 9.44 months. It was also revealed to be non-toxic, with an 87.95% viable cell percentage.

## Abstract 2

An ACL scaffold made of polycaprolactone (PCL)–hydroxyapatite (HA) and collagen is fabricated using an electrospinning method.

## Graphical abstract

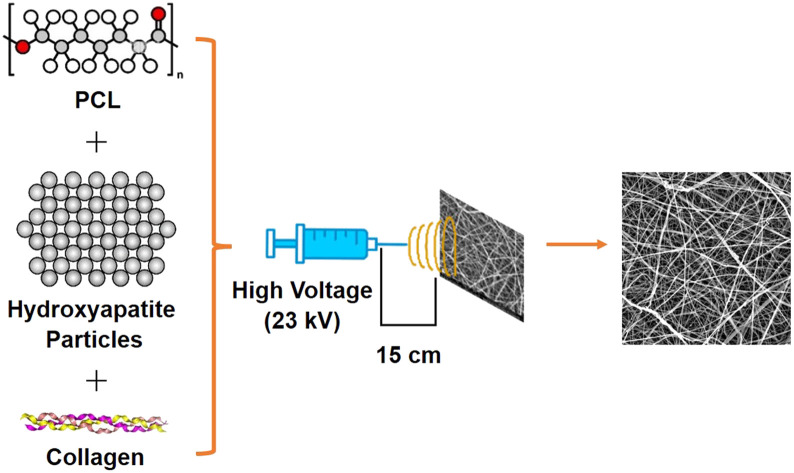

## 1. Introduction

Knee injuries are musculoskeletal system injuries that frequently occur alongside back injuries. This injury affects 48 per 1000 patients per year in Europe. In this instance, 9% of the ligaments, including the Anterior Cruciate Ligament, were damaged (ACL). Most ACLs rupture during sports activities. The incidence is highest between the ages of 15 and 25 in athletes who rotate the knee joint, such as those who play soccer, basketball, European handball, and volleyball. The injury is caused by valgus or external rotational trauma with a slightly bent knee.^1^ The severity of an ACL tear determines the treatment for the injury. If the injury can impair quality of life, especially in athletes who are actively moving, the treatment is ACL surgery. A torn ACL is typically treated with a graft harvested from the patient (autograft). However, autograft therapy poses a risk of tissue damage and tends to lengthen surgical procedures. Consequently, tissue engineering is utilized to develop an efficient method for ACL reconstruction.

In the last four decades, tissue engineering has emerged as an area of study. Tissue engineering aims to restore, maintain, or improve the function of damaged or lost tissues as a result of physiological, pathological, or mechanical conditions or trauma by developing biological replacements or reconstructing the tissues.^2^ Combining biomaterials such as scaffolds, stem cells, and growth factors will produce medically applicable tissue engineering products. The three factors, known as the tissue engineering triad, are inseparable: scaffold, stem cells, and growth factors. They resemble the regeneration of cells, tissues, and organs that occurs naturally.

Through the development of scaffolds, materials science has played a significant role in this process. The scaffold is a medium or framework that provides an environment for stem cells or other cells to adhere, proliferate, and differentiate, ultimately resulting in the formation of the desired tissue. The scaffold must be designed with the appropriate properties for its intended function, and its surface must have the correct morphology for cell attachment and differentiation.^3^

Engineered tissue for ACL injuries must possess the same biomechanical properties as the original ACL tissue in order to reconstruct the injury properly. Biopolymer is an excellent material that is frequently used to reconstruct damaged tissue. Biopolymers typically used for tissue reconstruction must also possess excellent biodegradability.^4^ However, biodegradable biopolymers must be carefully considered in terms of biocompatibility, as these properties can have toxic effects during degradation.^5^

One of the biopolymers employed in tissue engineering is polycaprolactone (PCL). With a modulus of elasticity between 0.21 and 0.44 GPa,^6^ PCL is very ductile and provides low stiffness. PCL is a polymer with excellent biocompatibility and degradation characteristics. PCL has a significantly lower rate of degradation than PLA, PGA, and PLGA.^7^ Two years are required for PCL to completely degrade.^8^ PCL has additional benefits, such as reducing local acidification and inflammation.^9^ Vascular, bone, cartilage, nerve, skin, and esophageal tissue are among the many applications of PCL in tissue engineering.^10^

To give PCL bioactive properties, hydroxyapatite must be added. Hydroxyapatite (HA) is the most abundant mineral in human bone. HA is frequently employed in biomedical implant applications or for tissue reconstruction and regeneration. HA possesses excellent bioactivity and osteoconductive properties. It is anticipated that the addition of HA to ACL reconstruction will stimulate cell growth in the femur and tibia, which are the scaffold's attachment sites so that the bone can integrate with the scaffold. HA is the most thermodynamically stable calcium phosphate ceramic compound in solution; its pH, temperature, and chemical composition are most similar to those of physiological fluids.^11^ In addition to HA, collagen must be added in order to match the Extracellular Matrix (ECM). Collagen has been used extensively to promote cell growth and differentiation during tissue formation. As the most abundant protein in the human body, collagen serves as physical support in tissues by occupying intercellular spaces, not only as structural support for regulating cells in connective tissue but also as a mobile, dynamic, and flexible substance that is essential for cellular behavior and network function.^3^ Collagen is also bio-inductive, possesses mechanical properties that are compatible with ECM, and is biodegradable, which makes it a popular choice for clinical applications. Multiple studies have demonstrated that collagen can enhance cell adhesion, promote bone cell proliferation, and enhance osteogenic cell differentiation. In addition, collagen dramatically increases the initial adhesion of the periosteal segment, which facilitates cell development and handling efficiency during implantation.

On the basis of the preceding information, the purpose of this study was to investigate the effect of variations in the composition of HA and collagen on the PCL–HA–collagen scaffold on a number of characteristics, including fiber surface morphology, fiber size, mechanical strength, degradation rate, and cytotoxicity. Electrospinning is used to create fibers because the ACL is anatomically composed of dense bands of collagen fibers. Electrospinning produces fibers with advantageous characteristics, such as high porosity, a large surface area, and continuous and quite long lengths.^12^

## 2. Materials and methods

**Fig. 1:**
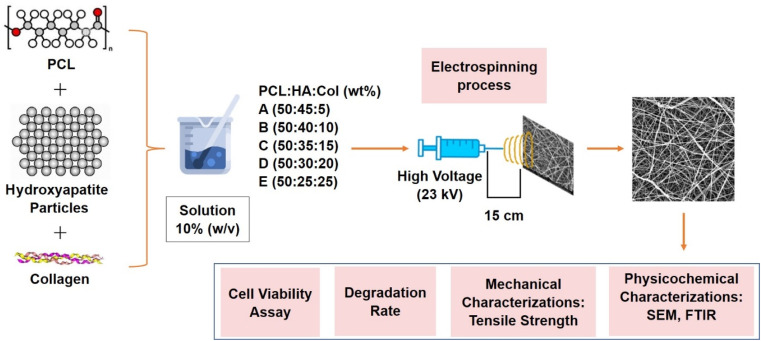
Schematic of methods to fabricate and characterize hydroxyapatite–polycaprolactone–collagen bone scaffold.

This study divided the production of fiber samples into three stages. The initial step involved the preparation of PCL–HA–collagen solutions of varying compositions. Stage 2 was the electrospinning process with constant process parameters for all samples, including a voltage of 23 kV, a needle-to-collector distance of 15 cm, and a flow rate of 2 mL h^−1^. The third stage was sample characterization, which included psychochemical characterization with FTIR spectrometer and Scanning Electron Microscope (SEM), mechanical properties, degradation rate, and cell viability evaluation using MTT assay. Fig. 1 provides a schematic representation of the study's work process.

### 2.1. Materials

The primary ingredients used in this study are polycaprolactone (PCL) from Sigma Aldrich (*M*_n_ = 80 000), hydroxyapatite (HA), and collagen (fish collagen). Chloroform and DMF from Merck, distilled water, Phosphate Buffered Saline (PBS) solution supplied by Oxoid, and 3-(4,5-dimethylthiazol-2-yl)-2,5-diphenyl tetrazolium bromide were used as solvents in the synthesis procedure (MTT). Electrospinning device from Genlab HK-7, Fourier transform infrared spectroscopy (FTIR-spectrophotometer) Shimadzu IRTracer-100, Scanning Electron Microscope (SEM) Hitachi FLEXSEM 1000, universal testing machine Shimadzu AGS 1kNX, and ELISA reader were utilized during the characterization process.

### 2.2. Methods

#### 2.2.1. Preparation of PCL–HA–collagen solution

The first step in creating scaffold fiber samples was to make a PCL–HA–collagen solution with the following composition variations: A (50 : 45 : 5), B (50 : 40 : 10), C (50 : 35 : 15), D (50 : 30 : 20), and E (50 : 25 : 25) (wt%) at a solution concentration of 10% (w/v). The PCL was dissolved in chloroform, the HA in distilled water, and the collagen in distilled water. A magnetic stirrer was used to mix and stir the respective solutions for 2 hours.

#### 2.2.2. Manufacturing of fiber through an electrospinning process

A 10 mL syringe was filled with the stirrer solution. The syringe was then connected to the electrospinning instrument. A flat-shaped collector was used in this electrospinning process. The fibers were collected using a flat collector covered in aluminum foil. The power supply's connecting cable was connected to the syringe needle. To ensure proper voltage transmission, the connecting wire must be properly attached to the needle.

In addition, fiber formation by electrospinning was carried out. The electrospinning process used a high voltage of 23 kV, a distance of 15 cm between the tip of the needle and the collector, and a flow rate of 2 mL h^−1^. The solution began to flow through the fibers that produce needles. The electric field influences the fibers' trajectory, causing them to deposit on the aluminum foil. The procedure was repeated until the syringe was empty. Electrospun fibers were the end result of the electrospinning process. They were cut to various sizes based on the requirements of the test.

### 2.3. Characterization

#### 2.3.1. Characterization of functional groups of PCL–HA–collagen scaffold using Fourier transform infra-red (FTIR) spectrophotometer

The electrospun fiber samples were then tested for functional groups using the Shimadzu IRTracer-100 FTIR at a wavenumber of 400–4000 cm^−1^. The FTIR test results show the relationship between % transmission and wavenumber spectrum (cm^−1^). The absorbance band formed in the sample's infrared spectrum was compared to standard data and the spectrum of the comparison compound was used for functional group analysis.

#### 2.3.2. Surface morphology and diameter of PCL–HA–collagen scaffold fiber using scanning electron microscope (SEM)

Surface morphology test of fiber samples using SEM Hitachi FLEXSEM 1000. The sample was gold-coated before measurements (Au). The fiber diameter was determined by analyzing SEM observation images with the ImageJ application. Calibration of the image pixel with the reference size should be the first step. The reference size is typically shown on the SEM image alongside a scale indicating the magnification level. The diameter of the average fiber can be measured using ImageJ software supported by Originlab to generate a sample diameter distribution plot. The light–dark area fraction is another parameter that can be analyzed microscopically from the surface structure. The dark fraction represents space, while the light area represents the formed fiber. To begin, the SEM image is segmented using a threshold to improve the definition of nanofiber and background. After adjusting the threshold, the area fraction analysis in question can be performed using Image-Histogram J's feature. The histogram shows a value of 0 for dark areas and a value of 255 for light areas.

#### 2.3.3. Measurement of mechanical properties of PCL–HA–collagen scaffold fiber

The mechanical test was performed to determine the sample's mechanical strength, specifically the modulus of elasticity, tensile strength, and elongation. Fiber samples were cut into dogbone shapes measuring 2 cm × 6 cm, and some were braided. Here we compare the design of scaffold using dogbone shapes (unbraided) and braided scaffold to understand in more detail the mechanical properties of the fabricated samples. It is predicted that the sample with dogbone and braided scaffold should have different in mechanical properties due to difference in how the scaffold receive a mechanical stress.^13^ A micrometer was used to measure the sample's thickness. The sample is then attached to the Shimadzu AGS 1kNX universal testing machine and a loading force is applied. The sample is then pulled until it breaks. Stress variations were measured during the tensile process. The slope of the linear region of the stress–strain curve at the maximum load-to-failure point was used to calculate tensile strength (N mm^−1^). Tensile strength characterization was performed three times on all unbraided and braided samples. The Ultimate Tensile Strength (UTS) value is calculated using eqn (1) and Young's modulus or modulus of elasticity (*E*) value is calculated using eqn (2), or it is calculated from the gradient value of the stress–strain linear curve and the elongation value using eqn (3).1
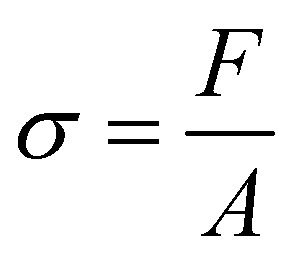
2
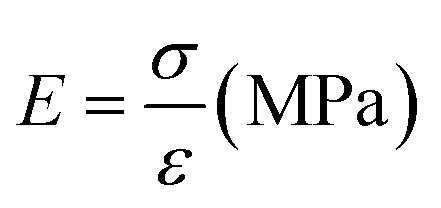
3
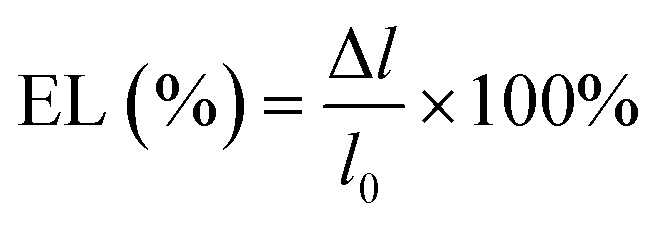
*σ* = stress (N m^−2^), *F* = force (N), *A* = surface area (m^2^), *ε* = strain (Δ*l*/*l*_0_), Δ*l* = change in length, *l*_0_ = initial length and EL = elongation (%).

#### 2.3.4. PCL–HA–collagen scaffold degradation test

This test was conducted to quantify the weight loss of each fiber sample after exposure to a Phosphate Buffer Saline (PBS) solution. Before being immersed in PBS (*W*_0_) solution, samples of fiber were weighed to determine their weight. The samples were submerged in a pH 7.4 PBS solution and incubated at 37 °C for 7, 14, 21, and 28 days. Following immersion, samples were weighed on days 7, 14, 21, and 28 to determine their final weight (*W*_t_). Each measurement was carried out thrice. Using eqn (4), the percentage of weight loss was determined. In addition, the degradation rate and estimates of the completely degraded scaffold in the solution are calculable.4
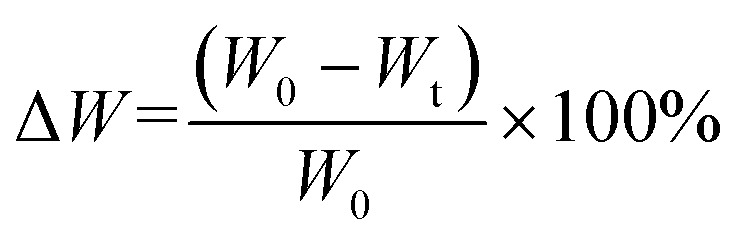


#### 2.3.5. Cytotoxicity test for PCL–HA–collagen scaffold

A cytotoxicity test is a test performed to determine the levels of toxicity in prepared samples. This evaluation employs fibroblast cell culture (cell line BHK 21). The cells were incubated on Eagle's medium, or until a single confluent layer formed on the respective wall. Thus, the cells seeded onto scaffold for 24 hours. The remaining serum was then cleaned with dimethyl sulfoxide (DMSO) after the removal of the medium. To prevent colonization, the cells were then separated using 0.25 percent vergence trypsin.

In addition, Eagle media cells and MTT reagent were added, then transferred to a microplate 96 and incubated at 37 °C for four hours. Each measurement was performed thrice. To stop the reaction, DMSO was added to each well, which was then vortexed for 5 minutes to achieve homogeneity. Repeating each sample four times. With the aid of an ELISA reader, the optical density (OD) was determined. Eqn (5) was used to calculate cell viability.5
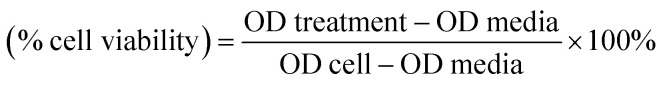


## 3. Results

**Fig. 2:**
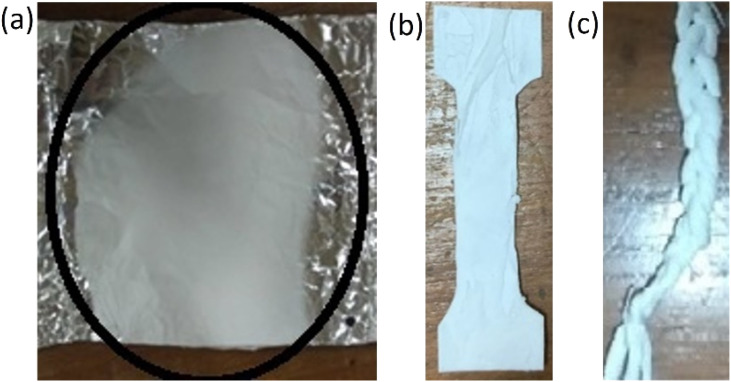
Fiber electrospinning process (a) fiber on aluminum foil (b) dogbone fiber and (c) braided fiber for mechanical testing.

Fig. 2a depicts the resultant fiber of the electrospinning process. The fibers from the aluminum foil were removed. It was then sized appropriately for the various tests. Fibers were separated for mechanical property tests (Fig. 2b). Fig. 2c demonstrates the braided fibers.

### 3.1. Analysis of the PCL–HA–collagen scaffold functional groups from the Fourier transform infra-red (FTIR) spectrum

**Fig. 3:**
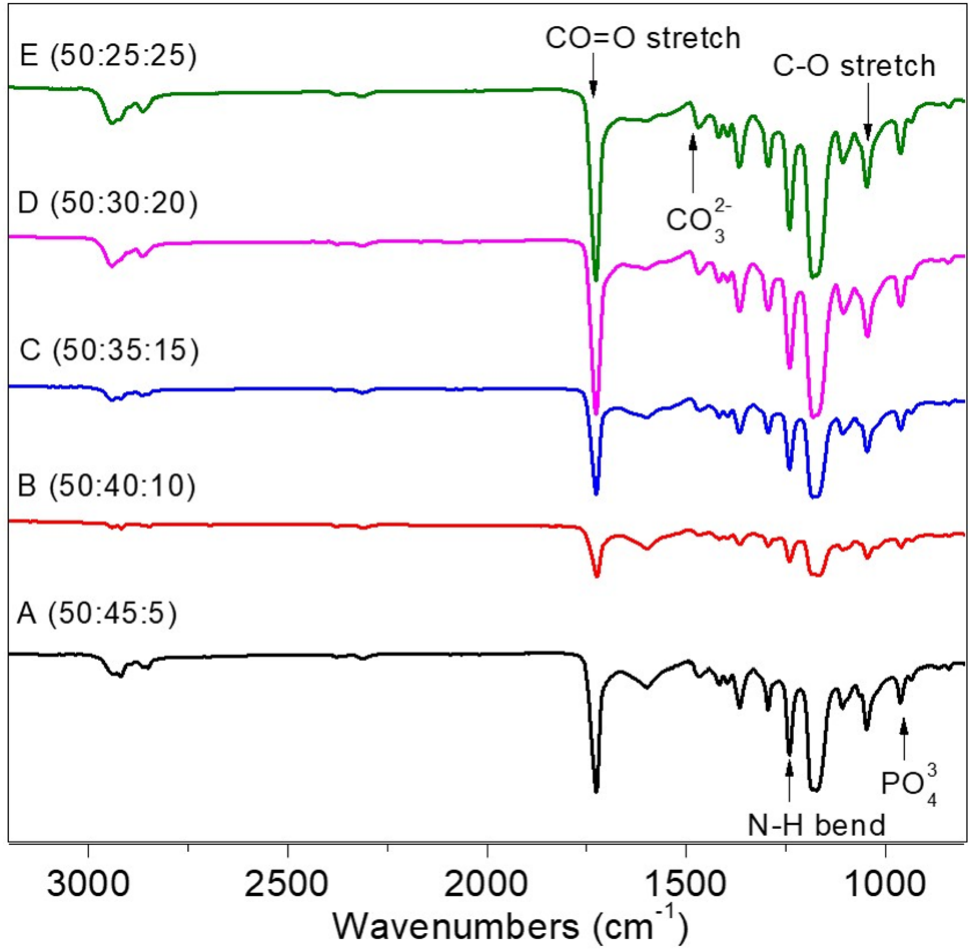
FTIR spectrum results of PCL : HA : collagen scaffold.

The interaction of infrared radiation and molecular vibrations produces the Fourier Transform Infra-Red (FTIR) spectrum. Each type of molecular vibration has a unique value expressed by a wavenumber (cm^−1^). Fig. 3 depicts the PCL–HA–collagen scaffold spectrum. Table 1 shows the identification of functional groups that appear in each sample. The presence of a C=O stretch group in the wave number region 1724.29–1724.36 cm^−1^ served as the identifier for PCL. In addition, the presence of the C–O stretch at a wavenumber of 1105.21–1107.14 cm^−1^ indicates PCL characteristics. The hydroxyapatite characteristic was the PO_4_^3−^ group at wavenumber 960.55 cm^−1^.^14^ Additional markers include the CO_3_^2−^ group at wave numbers 1415.75–1417.68 and 1463 cm^−1^.^14^ In addition, the collagen marker is identified by an amide III group (N–H bend) in the wave number region of 1240.23 cm^−1^.

**Table 1:** Functional group analysis and wavenumbers

Functional group	Bonds	Standard value wavenumber (cm^−1^)	Wavenumber (cm^−1^)				
			Sample A	Sample B	Sample C	Sample D	Sample E
Ester carbonyl group within PCL	C=O stretch	1730–1700	1724.36	1724.36	1726.29	1726.29	1726.29
	C–O stretch	1260–1000	1107.14	1105.21	1107.14	1105.21	1105.21
Collagen, amide II	N–H bend	1550 and 1500	1240.23	1240.23	1240.23	1240.23	1240.23
Inorganic ions in HA	PO_4_^3−^	1100–900	960.55	960.55	960.55	960.55	960.55
	CO_3_^2−^	1490–1410/880–860	1415.75	1463	1417.68	1417.68	1417.68

### 3.2. Analysis of the morphology and diameter of the fiber from the scanning electron microscope (SEM)

**Fig. 4:**
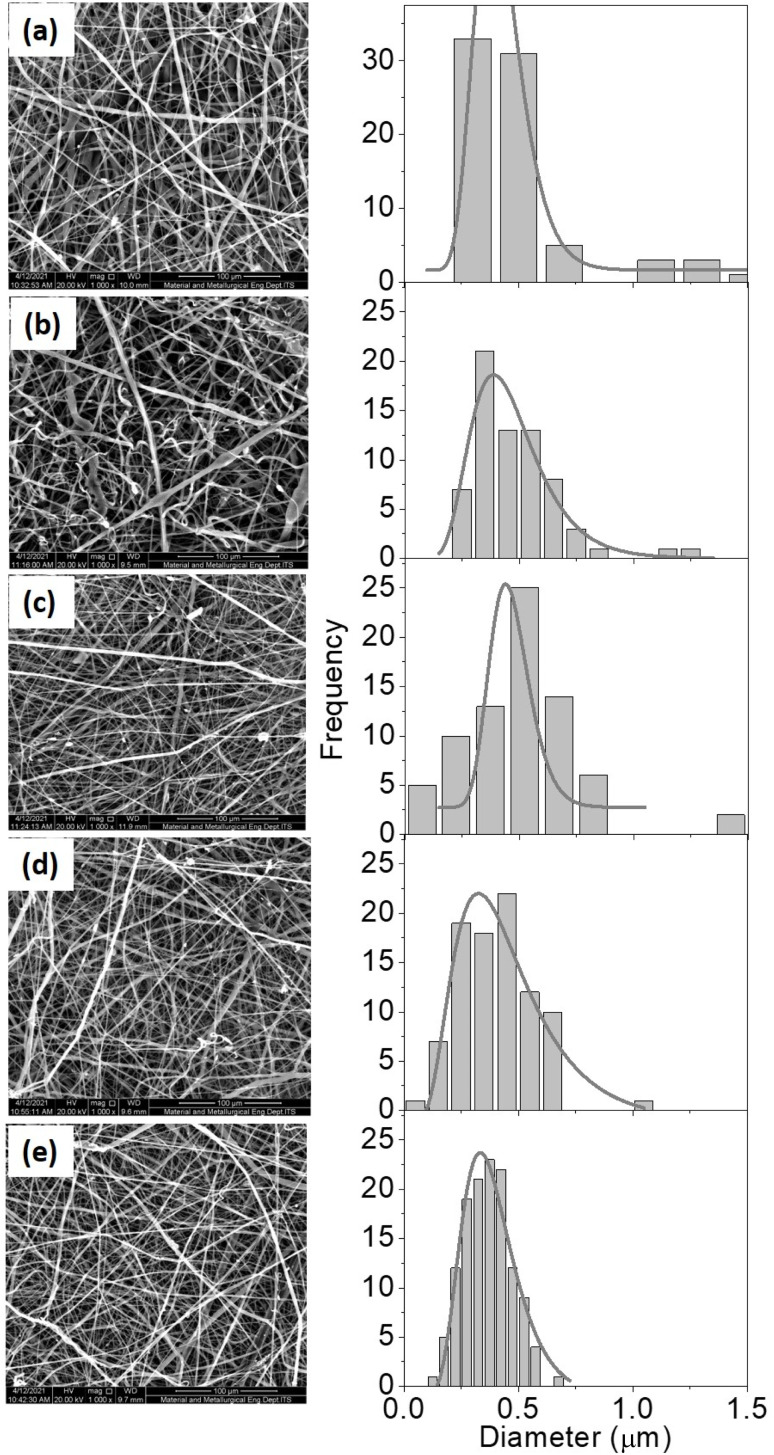
SEM results (right) and fiber diameter distribution histogram (left) for samples with different ratio of PCL : HA : collagen; A (50 : 45 : 5), B (50 : 40 : 10), C (50 : 35 : 15), D (50 : 30 : 20), and E (50 : 25 : 25).

The fiber surface morphology was examined using a 1000× magnification Scanning Electron Microscope (SEM) (Fig. 4). Because the collector used is not drum-shaped, the fibers tend to pile up and arrange themselves randomly in all samples. Furthermore, it can be caused by the solution being exposed to high voltages that are not optimally attracted, causing instability and random orientations. The shape of the aligned fiber is more advantageous than that of the random fiber. Fibers with aligned shapes promote cell proliferation and improve sample mechanical properties.^15^ Furthermore, all samples had rough surface morphology. This could be because hydroxyapatite and collagen were added. According to Han's 2015 research, the morphology of PCL samples with hydroxyapatite and collagen was coarser than PCL without hydroxyapatite and collagen.^16^ The formation of beads in Fig. 4 can be attributed to the solution's low dielectric properties. DMF solvent can increase the dielectric properties, preventing the formation of beads.

Based on Fig. 4, the diameter of the fibres is presented in Table 2.

**Table 2:** Average of fiber diameter for various PCL : HA : collagen composition

Sample name	PCL : HA : collagen composition (wt%)	Fiber diameter range (nm)	Average fiber diameter (nm)
A	50 : 45 : 5	203–1535	488 ± 271
B	50 : 40 : 10	201–1210	482 ± 192
C	50 : 35 : 15	145–1047	433 ± 154
D	50 : 30 : 20	114–1078	401 ± 162
E	50 : 25 : 25	145–689	365 ± 104

Table 2 revealed that the fiber diameter varied, with the diameter decreasing as the collagen content increased. The light–dark area fraction was also determined using SEM analysis that we have tabulated in Table 3.

**Table 3:** Dark–light fraction

Sample	PCL : HA : collagen composition (wt%)	Dark fraction (%)	Light fraction (%)
A	50 : 45 : 5	56.43	43.57
B	50 : 40 : 10	57.40	42.60
C	50 : 35 : 15	59.72	40.28
D	50 : 30 : 20	60.91	39.09
E	50 : 25 : 25	64.15	35.85

The value of the dark fraction represented an area completely void of fiber. This value was affected by the collagen content percentage of the scaffold. Collagen is essential for hydroxyapatite binding.^17^ Due to the high collagen concentration in the composite solution, the collagen does not perfectly combine with the hydroxyapatite. Greater the collagen concentration in a solution, the smaller the diameter produced (Table 2). Because collagen is a polyelectrolyte or an ionized linear polymer with a large number of functional groups, its presence can increase the conductivity of polymer solutions. The resulting fiber will be more delicate and have a smaller diameter as the conductivity of the solution increases. When the fiber diameter decreases, the fibers are oriented more randomly, resulting in a lower fiber density and a larger empty area (Table 3).

### 3.3. Mechanical properties of PCL–HA–collagen fiber scaffold

**Fig. 5:**
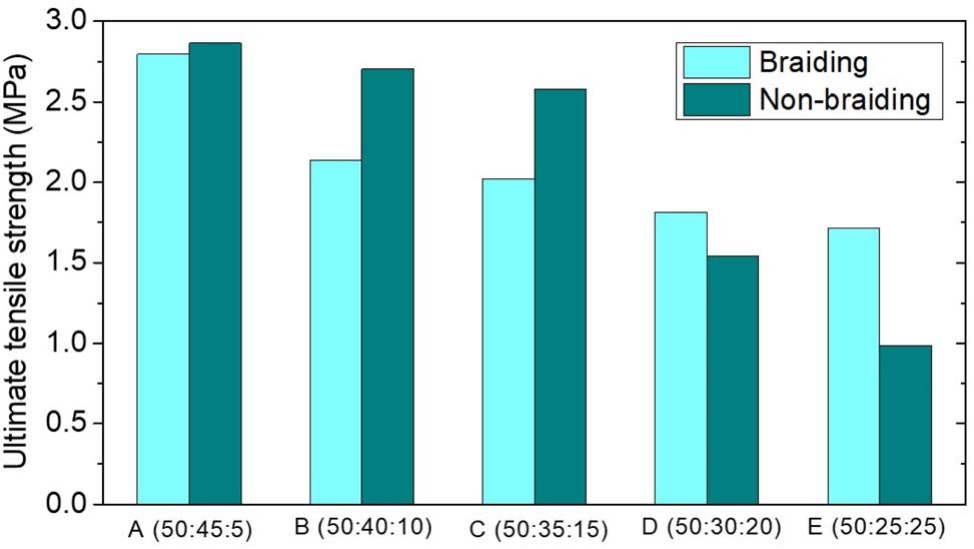
UTS of PCL–HA–collagen scaffold.

**Fig. 6:**
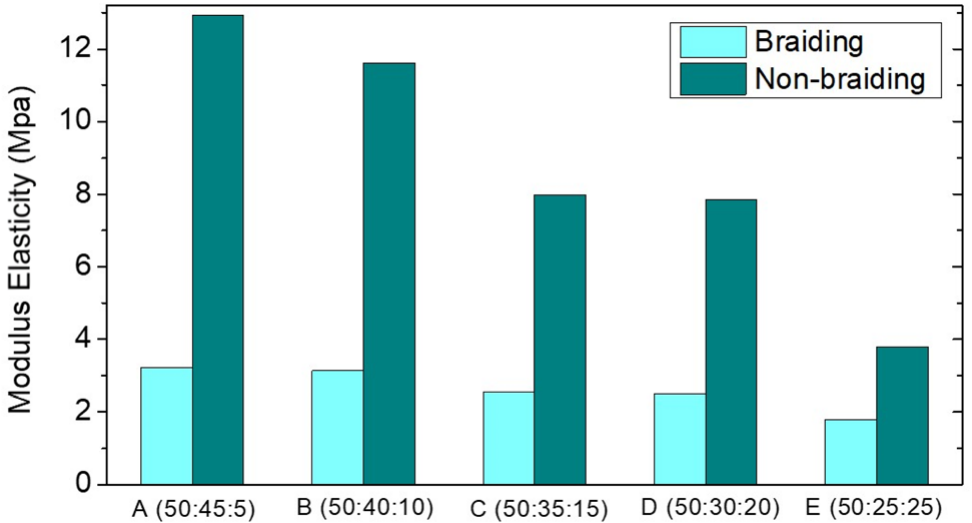
Elastic modulus of PCL–HA–collagen scaffold.

**Fig. 7:**
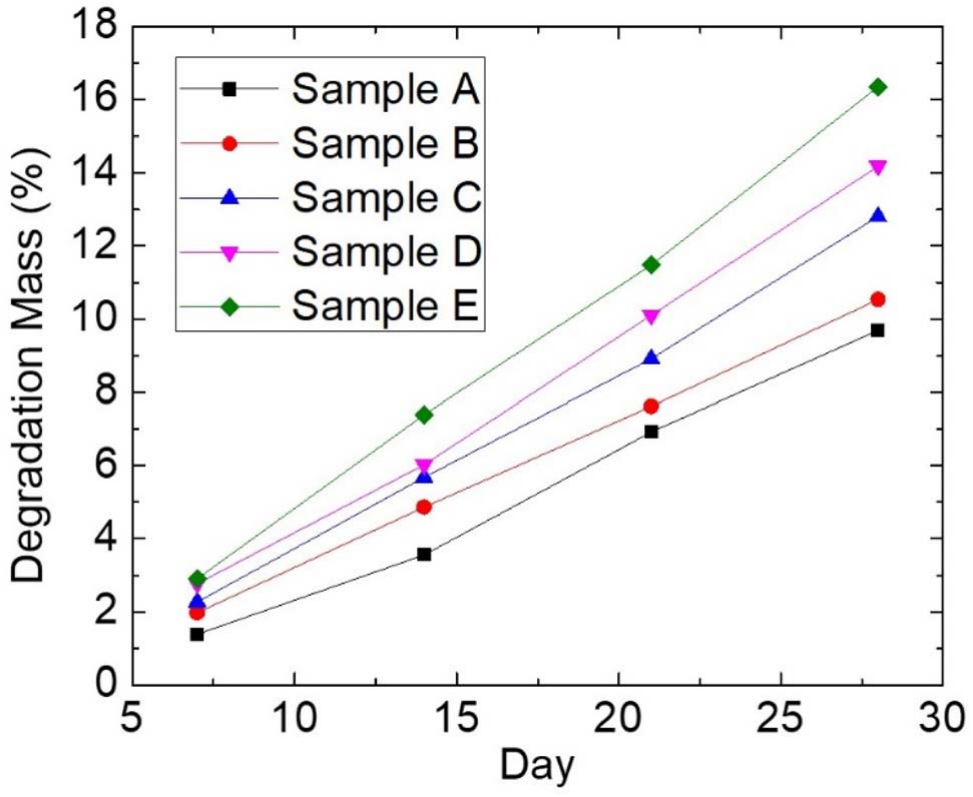
Mass degradation of PCL–HA–collagen scaffold sample.

Mechanical properties were determined on both braided and non-braided samples. Table 4 shows the values of Ultimate Tensile Strength (UTS), modulus of elasticity, and elongation in the samples. The lower the UTS value and the higher the elastic modulus, the lower the HA composition and the higher the collagen composition. The ultimate tensile strength of ACL is approximately 36 MPa.^18^ Furthermore, the modulus of elasticity of ACL ranged from 65 to 111 ± 29 MPa.^19^ The UTS value and modulus of elasticity do not meet the human ACL standard, as shown in Fig. 6 and 7. This could be due to the random orientation of the fibers. Randomly oriented fibers cannot withstand stress in the same direction, resulting in low mechanical strength.^20^ In the braiding sample, the UTS value and modulus of elasticity were lower. This could be due to the braiding process being done by hand, resulting in less tight braids; as a result, the mechanical strength was lower than that of the non-braiding one (Fig. 5).^21^

**Table 4:** The mechanical properties of the PCL–HA–collagen scaffold samples

Sample name	PCL : HA : collagen composition (wt%)	Braiding			Non Braiding		
		Ultimate tensile strength (UTS) (MPa)	Strain	Young's modulus (MPa)	Ultimate tensile strength (UTS) (MPa)	Strain	Young's modulus (MPa)
A	(50 : 45 : 5)	2.79	1.25	3.22	2.86	1.49	12.94
B	(50 : 40 : 10)	2.14	1.42	3.13	2.70	1.34	11.61
C	(50 : 35 : 15)	2.02	1.62	2.54	2.58	1.29	7.99
D	(50 : 30 : 20)	1.81	1.72	2.49	1.54	1.21	7.84
E	(50 : 25 : 25)	1.71	1.69	1.79	0.99	0.92	3.79

### 3.4. PCL–HA–collagen scaffold degradation rate

One of the most important factors in tissue engineering is the rate of degradation. To determine whether the scaffold is still available or has been completely degraded during the tissue growth process, the degradation rate must be measured. Fig. 7 shows the results of the mass of the degraded samples (in %) after 7, 14, 21, and 28 days.

According to the linear regression results in Fig. 7, the rate of degradation of each sample corresponds to the gradient value of each regression equation. Based on the regression equation, it is also possible to predict when the entire scaffold will be completely degraded, assuming a constant degradation rate. Table 5 displays the results of the calculation of the degradation rate and the estimated time-out for each sample. The degradation rate of the scaffold must correspond to the formation of ligaments. The rate of cell proliferation will be disrupted if degradation occurs too rapidly. In contrast, if the rate of degradation is too slow, it will interfere with the tissue's biological function.^22^

**Table 5:** The results of the calculation of the degradation rate and the estimated time-out of the PCL–HA–collagen scaffold

PCL : HA : collagen composition (wt%)	Sample	Degradation rate (g per day)	Estimated degradation time (months)
50 : 45 : 5	A	6 × 10^−5^	9
50 : 40 : 10	B	8 × 10^−5^	9
50 : 35 : 15	C	9 × 10^−5^	7
50 : 30 : 20	D	1 × 10^−4^	6
50 : 25 : 25	E	1 × 10^−4^	6

### 3.5. PCL–HA–collagen scaffold cell viability

**Fig. 8:**
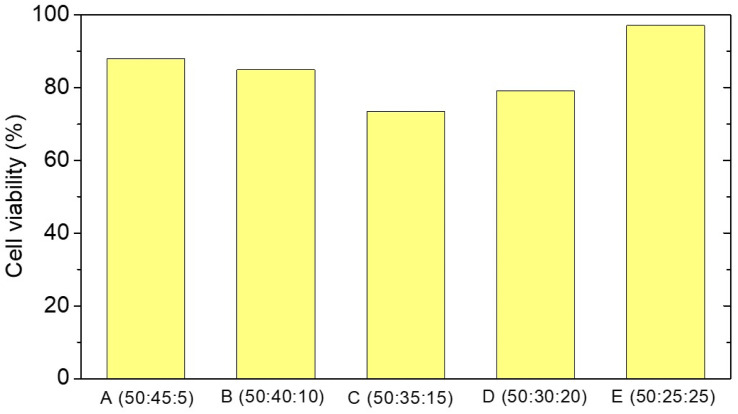
Average cell viability of PCL–HA–collagen scaffold samples.

As part of tissue engineering, a scaffold must be non-toxic. Using Baby Hamster Kidney (BHK-21) cells, a cytotoxicity test was conducted using the MTT assay method. The MTT assay is based on the principle that cells with metabolic activity reduce MTT salts through the work of enzymes. When the enzyme reacts with MTT, a purple formazan will be produced. The absorbance of living cells will be determined by spectrometry measurements of the intensity of this purple hue. Fig. 8 illustrates the results of the live cell count. Sample E with a PCL–HA–collagen ratio of 50 : 25 : 25 exhibited the highest cell viability. This result is the result of multiple factors; in the sample, the addition of collagen, which can facilitate fibroblast growth and tissue regeneration, allows BHK cells to perform cell activity more effectively.

## 4. Discussion

The FTIR spectrum revealed the presence of PCL, hydroxyapatite, and collagen marker groups based on functional group analysis. Because the FTIR spectrum results do not offer any new functional groups apart from the functional groups of the three materials, these three materials are only physically mixed. The functional groups are the HA PO_4_^3−^ a group, the collagen amide III (N–H bend), and the carbonyl group of the C=O stretch group. As a result, the synthesis of PCL–HA–collagen scaffold in the form of fiber *via* the electrospinning process was deemed successful.^16^

PCL samples containing hydroxyapatite and collagen had a rougher surface morphology than PCL samples lacking hydroxyapatite and collagen. Further analysis using ImageJ software revealed that the average fiber diameter ranged between 365 and 488 nm (Table 2). Nanostructures have a high surface area ratio, allowing for more cell attachment space than other structures. Furthermore, fibers with a diameter of 1000 nm can increase the activity of cells in forming an extracellular matrix.^23^

Mechanical strength is an important property of scaffolding, especially for ACL. The ACL is a ligament that acts as a back and front movement barrier as well as a knee stabilizer. To perform this function, you must have a high modulus of elasticity and a low UTS. Similarly, one of the parameters that must be considered on the ACL scaffold is the mechanical property. Mechanical strength (modulus of elasticity and UTS) increased with increasing hydroxyapatite composition in PCL : HA : collagen scaffold samples. However, the results are still insufficient to match the mechanical strength of the human ACL. The addition of hydroxyapatite to collagen can increase the scaffold's modulus of elasticity and UTS.^24^ Braiding approach should be able to improve the mechanical properties of the scaffold.^13,25^ However, our braiding process has failed to increase the mechanical strength value. This is presumably due to the fact that the process is done manually. Therefore, one bond is not perfectly interwoven with another, in contrast with previous report of braided scaffold fabricated using braiding machine to form a perfect braided scaffold.^26^

The presence of collagen in the sample causes the degradation rate to be faster, resulting in a higher quality of mass degraded in the sample. This is due to the fact that collagen interacts with water more easily than PCL and HA. Furthermore, collagen is a polymer with amorphous properties. Ligaments can regenerate for a period of 6–8 months. As a result, in this study, the appropriate degraded samples were C, D, and E.^27^

A live cell percentage of more than 60% is required for tissue engineering.^28^ The samples in this study had a percentage value of living cells above 60%, indicating that the sample does not have toxic properties. Based on the results of the above characterizations, the PCL : HA : collagen fiber scaffold has the potential as an ACL scaffold. However, mechanical strength needs to be increased.

## 5. Conclusion

Variations in HA and collagen composition influence fiber diameter and morphology, porosity, and mass loss percentage. The samples with a high hydroxyapatite concentration still contained beads. As the concentration of collagen increases, no beads form. As the average diameter of all samples is less than 1000 nm, cell attachment is facilitated. The percentage of mass lost increases as collagen levels rise. The sample is non-toxic in the cytotoxicity test because the percentage of viable cells is greater than 60%. The optimal composition is found in samples with a ratio of 50 : 45 : 5 PCL : HA : collagen. This sample's fiber diameter ranges from 203 to 1535 nm, with a mean fiber diameter of 488 ± 271 nm. The UTS value and modulus of elasticity for the braided sample were 2.796 MPa and 3.224 MPa, whereas they were 2.864 MPa and 12.942 MPa, respectively, for the unbraided sample. They estimated a total of 9.44 months of mass exhaustion. It contains 87.95% of living cells and is non-toxic.

## Conflicts of interest

The authors declare no conflict of interest.

## Supplementary Material
